# The *CFTR* Met 470 Allele Is Associated with Lower Birth Rates in Fertile Men from a Population Isolate

**DOI:** 10.1371/journal.pgen.1000974

**Published:** 2010-06-03

**Authors:** Gülüm Kosova, Joseph K. Pickrell, Joanna L. Kelley, Patrick F. McArdle, Alan R. Shuldiner, Mark Abney, Carole Ober

**Affiliations:** 1 Committee on Genetics, Genomics and Systems Biology, University of Chicago, Chicago, Illinois, United States of America; 2 Department of Human Genetics, University of Chicago, Chicago, Illinois, United States of America; 3 Department of Medicine, University of Maryland School of Medicine, Baltimore, Maryland, United States of America; 4 Geriatrics Research and Education Clinical Center, Veterans Administration Medical Center, Baltimore, Maryland, United States of America; 5 Department of Obstetrics and Gynecology, University of Chicago, Chicago, Illinois, United States of America; Princeton University, United States of America

## Abstract

Although little is known about the role of the cystic fibrosis transmembrane regulator (*CFTR*) gene in reproductive physiology, numerous variants in this gene have been implicated in etiology of male infertility due to congenital bilateral absence of the vas deferens (CBAVD). Here, we studied the fertility effects of three CBAVD–associated *CFTR* polymorphisms, the (TG)m and polyT repeat polymorphisms in intron 8 and Met470Val in exon 10, in healthy men of European descent. Homozygosity for the Met470 allele was associated with lower birth rates, defined as the number of births per year of marriage (*P* = 0.0029). The Met470Val locus explained 4.36% of the phenotypic variance in birth rate, and men homozygous for the Met470 allele had 0.56 fewer children on average compared to Val470 carrier men. The derived Val470 allele occurs at high frequencies in non-African populations (allele frequency  = 0.51 in HapMap CEU), whereas it is very rare in African population (Fst  = 0.43 between HapMap CEU and YRI). In addition, haplotypes bearing Val470 show a lack of genetic diversity and are thus longer than haplotypes bearing Met470 (measured by an integrated haplotype score [iHS] of −1.93 in HapMap CEU). The fraction of SNPs in the HapMap Phase2 data set with more extreme Fst and iHS measures is 0.003, consistent with a selective sweep outside of Africa. The fertility advantage conferred by Val470 relative to Met470 may provide a selective mechanism for these population genetic observations.

## Author Summary

Cystic fibrosis (CF) is the most common lethal recessive disorder in European-derived populations and is characterized by clinical heterogeneity that involves multiple organ systems. Over 1,600 disease-causing mutations have been identified in the cystic fibrosis transmembrane regulator (*CFTR*) gene, but our understanding of genotype–phenotype correlations is incomplete. Male infertility is a common feature in CF patients; but, curiously, CF–causing mutations are also found in infertile men who do not exhibit any other CF–related complications. In addition, three common polymorphisms in *CFTR* have been associated with infertility in otherwise healthy men. We studied these three polymorphisms in fertile men and show that one, called Met470Val, is associated with variation in male fertility and shows a signature of positive selection. We suggest that the Val470 allele has risen to high frequencies in European populations due a fertility advantage but that other genetic and, possibly, environmental factors have tempered the magnitude of these effects during human evolution.

## Introduction

The cystic fibrosis transmembrane conductance regulator (*CFTR*; OMIM 602421) gene functions as a chloride channel that regulates salt and water transport across epithelial cell membranes. More than 1,600 mutations (Cystic Fibrosis Mutation Database; http://www.genet.sickkids.on.ca/cftr/) in the *CFTR* gene cause cystic fibrosis (CF; OMIM 219700), an autosomal recessive disorder affecting the exocrine glands of the respiratory, digestive and reproductive systems. The clinical manifestations of CF in affected individuals vary widely, with both age at diagnosis and lethality ranging from the first year of life to the third (and later) decade [1]. One symptom, however, that is present in nearly all male CF patients is infertility due to congenital bilateral absence of the vas deferens (CBAVD; OMIM 277180), which results from blockage in the transport of spermatozoa from testicular tissues to the distal genital track [2]. Curiously, CBAVD is also a cause of infertility in otherwise healthy men, accounting for ∼2% of all male infertility cases. However, 80% of men with isolated CBAVD carry one or two mutations in the *CFTR* gene [3], defining a primarily genital form of CF.

The most common genetic cause of CBAVD is compound heterozygosity for a 5-thymidine (5T) repeat allele at the 3′ splice acceptor site of intron 8 and a CF-causing mutation in the *CFTR* gene [3]. The length of the polyT tract within intron 8 is associated with splicing efficiency of exon 9 [4]. The shorter 5T allele, compared to the more common 7T or 9T alleles, results in under-utilization of the splice site and increased proportions of CFTR transcripts lacking exon 9, which encode a nonfunctional protein. However, the 5T allele alone does not explain all cases of CBAVD. Other polymorphisms, including a TG repeat [(TG)m] located immediately upstream of the polyT tract in intron 8, and an amino acid changing polymorphism (Met470Val; 1540A>G [rs213950]) in exon 10, have also been implicated [5], [6]. For example, longer TG repeat alleles (TG12 or TG13) alter the stability of the mRNA secondary structure and decrease exon 9 splicing efficiency, thereby increasing the penetrance of the 5T allele [7], [8]. Likewise, a valine at a common polymorphism at amino acid 470 (Val470) results in the CFTR protein to mature more quickly, but with lower activity compared to the methionine (Met470) allele [8]. An association between the 5T and Val470 alleles in men with CBAVD but not in fertile controls led de Meeus et al. to suggest that the Met470Val locus acts as a modifier by increasing the penetrance of the 5T allele in CBAVD [5].

To further investigate the contribution of *CFTR* polymorphisms in male reproduction, we examined the effects of the intron 8 (TG)m and polyT variants and the Met470Val polymorphism on the variation in natural fertility in healthy men. We conducted this study in the Hutterites, a founder population of European descent [9], [10]. The Hutterites provide many advantages for genetic studies of fertility. First, they practice a communal lifestyle that minimizes variation in socioeconomic, cultural, religious, and other factors that might affect reproductive practices. For example, contraceptive use is limited and a desire for large families is widespread. As a result, Hutterite family sizes are large (median completed family size >10 in 1960's [11], [12]) and reproductive rates are among the highest observed in humans [13]. Although the overall allelic architecture in the Hutterites is similar to that of other European populations [14], [15], there are only two CF-causing mutations segregating in the Hutterites, ΔF508 and the more common, Hutterite-specific M1101K. We previously examined the effects of carrier status for these two mutations on family size and birth rate in nearly the same men considered in this study, but found no association with reproductive outcomes [16]. The results we report here, however, suggest a significant contribution of at least one common *CFTR* polymorphism to natural variation in male fertility, and provide further support for the role of this protein in normal male reproductive processes.

## Results/Discussion

We genotyped 204 married Hutterite men for the Met470Val and intron 8 polyT and (TG)m repeat polymorphisms; allele and haplotype frequencies are shown in Table 1 and Table 2. The CBAVD-associated 5T, TG12, and TG13 alleles are either absent (5T, TG13) or rare (TG12) in the Hutterites. On the other hand, the Val 470 allele occurs at high frequency (frequency 0.29). The Val allele resides on two haplotypes in the Hutterites, one common (TG11-7T-Val470) and one rare (TG12-7T-Val470).

**Table 1:** Frequencies of the *CFTR* polymorphisms.

Polymorphism	Sample size	Alleles	Allele frequencies
(TG)m	203	10/11/12	0.52/0.43/0.05
polyT	203	5/7/9	0.00/0.91/0.09
Met470Val^1^	204	Met/Val	0.71/0.29
Met470Val^2^	315	Met/Val	0.35/0.65

1 Allele frequencies in the Hutterite men.

2 Allele frequencies in the Amish men.

**Table 2:** Frequencies of the *CFTR* haplotypes in the Hutterites.

Haplotype	Frequency
TG10 – 7T – Met470	0.45
TG11 – 7T – Met470	0.15
TG12 – 7T – Met470	0.03
TG10 – 9T – Met470	0.08
TG11 – 9T – Met470	0.01
TG11 – 7T – Val470	0.26
TG12 – 7T – Val470	0.02

To assess the effects of these polymorphisms on male fertility, we defined a measure of “birth rate” as the number of births per year of marriage in men with at least two children (see Materials and Methods). Associations between the *CFTR* alleles and haplotypes and birth rate were examined in Hutterite men using a regression-based test designed for large complex pedigrees, and which corrects for the relatedness between all pairs of men in this study [17]. The results of the association studies are summarized in Table 3.

**Table 3:** Results of association tests with birth rate in Hutterite men.

Locus	Allele associated with lower fertility	*P*-value	% phenotypic variance explained
Met470Val (model 1)^1^	Met470	0.0096	4.59
Met470Val (model 2)^2^	Met470	0.0029	4.36
(TG)m	TG10	0.126	1.87
polyT	9T	0.060	1.78

1 All three genotypes were tested individually.

2 People carrying Met/Val and Val/Val genotypes were combined and tested against Met/Met homozygotes.

**Figure 1:**
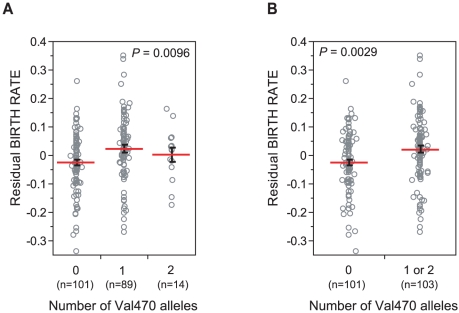
*CFTR* Met470Val genotypes and birth rate in Hutterites men. The residuals of birth rate, corrected for relatedness is shown on the y-axis, and the number of Val470 alleles at the *CFTR* Met470Val locus is shown on the x-axis. Sample sizes for each genotype group are shown under x-axis. Each grey circle corresponds to an individual subject. Red horizontal lines show the means and black whiskers show the standard errors. (A) Met470Val genotype (Model 1); grouped by 0 (Met/Met), 1 (Met/Val) or 2 (Val/Val) copies of the Val allele; (B) Met470Val genotype (Model 2) with Val/Val and Met/Val men combined.

Homozygosity for the Met470 allele was associated with significantly lower birth rates in Hutterite men (*P* = 0.0096; Figure 1A), and accounted for 4.59% of the residual variance (after adjusted for covariates, see Materials and Methods) in birth rate between males. The association remained significant when Val470 homozygotes (N = 14) and heterozygotes (N = 89) were combined (Model 2), consistent with a recessive effect of the Met470 allele on increased birth rates (*P* = 0.0029; Figure 1B). Both models remain significant after adjusting for multiple comparisons by a conservative Bonferroni correction (Model 1 *P*_c_ = 0.038, Model 2 *P*_c_ = 0.012). There was no significant association between alleles at the (TG)m locus and birth rate. A marginal association was observed between the 7T allele and increased birth rates (7T vs. 9T, *P* = 0.060), but we attribute this to linkage disequilibrium (LD) with the Val470 allele (D' = 1.0, Table 2).

Nine men in this sample carried the M1101K mutation (none were ΔF508 carriers). In all cases, the M1101K mutation was on the Met470 background. Therefore, to remove the potential confounding effects of M1101K, we repeated our analyses after excluding these nine men. The association with Met470Val remained equally significant (Model 1 *P* = 0.0059, Model 2 *P* = 0.0020), suggesting that the observed fertility effects associated with Met470Val are not due to this pathogenic *CFTR* mutation.

**Figure 2:**
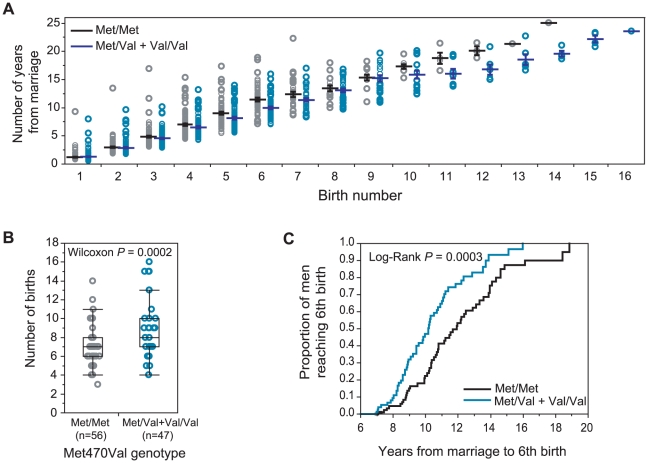
Met470Val genotypes and fertility in Hutterite men. (A) Cumulative plot of the number of years from marriage (y-axis) to each birth (x-axis) by genotype. Black horizontal lines show the means and whiskers show standard errors. (B) Box plot and distribution of the number of births (y-axis) among men by genotype (x-axis) for men who are married at least 11.5 years (mean number of years from marriage to last birth for the men in this sample). (C) Survival curves showing the proportion of men reaching the 6^th^ birth (equal to the mean and median of number of births among men in this sample) (y-axis) by 6 to 20 years of marriage (x-axis) by genotype. The distributions of total length of marriages are similar for men in both genotype groups (not shown).

On the other hand, the association with the Met470Val locus in Hutterite men is quite robust. Figure 2A shows the cumulative distribution of the number of years from marriage to each birth by genotype. On average, Met/Met men achieve each birth in more time than men with one or two copies of the Val allele. The difference between the means of the genotype groups increases with increasing birth number, reflecting a cumulative, positive effect of the Val allele (relative to Met/Met) on male fertility. The average effect of homozygosity for the Met470 allele compared to carrying one or two copies of the Val470 is a decrease of 0.049 births per year of marriage (Figure 1B). This corresponds to 0.56 fewer births over the course of an average reproductive period (11.5 [±5.0] years in this cohort). For example, Met470 homozygous men who are married 11.5 years or longer have a median of 7 children compared to 8 children in Val470 carrier men (Wilcoxon *P* = 0.0002; Figure 2B). Finally, the time required to achieve 6 births (the overall mean and median family size in our sample) is significantly longer for Met470 homozygotes (Figure 2C). The median time to having a sixth child is 11.9 years (upper, lower quartiles: 10.2, 14.0) among Met/Met men and 10.18 years (upper, lower quartiles: 8.7, 11.9) among Met/Val+Val/Val men (Log-rank *P* = 0.0003).

We next attempted to replicate this association in another population that is also characterized by high natural fertility rates and large families, the Old Order Amish of Lancaster County, Pennsylvania [18]. Three hundred fifteen Amish men, for whom reproductive histories were available, were genotyped for the Met470Val polymorphism. In this Amish population, the derived Val allele is the major allele, with a frequency of 0.65. As a result, only 37 men were homozygous for the Met allele. Consistent with results in the Hutterites, Met/Met men had lower birth rates (0.46±0.13 births/year) than Met/Val (0.50±0.14 births/year) or Val/Val (0.49±0.17 births/years) men (Table 4). This difference, however, was not statistically significant (*P* = 0.22), most likely due to the small number of Met/Met homozygous men, and the corresponding lack of power. In addition, (TG)m and polyT genotypes were not available in the Amish population. Therefore, we can not rule out possible interactions with the haplotype background or independent effects of these repeat polymorphisms, especially if their allele frequencies are notably different from the Hutterites, as in the case of Met470Val polymorphism.

**Table 4:** Birth rates in Amish men by Met470Val genotypes (*P* = 0.22).

Genotype	N	Mean (± SD)
Met/Met	37	0.46±0.13
Met/Val	147	0.50±0.14
Val/Val	131	0.49±0.17

**Figure 3:**
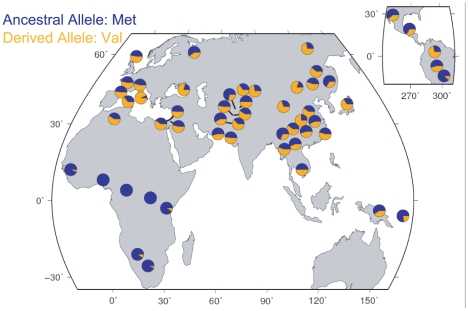
Geographic distribution of the Met470Val polymorphism in HGDP samples. The relative frequencies of each allele are shown as blue (ancestral Met470 allele) and orange (derived Val470 allele) pie slices.

**Figure 4:**
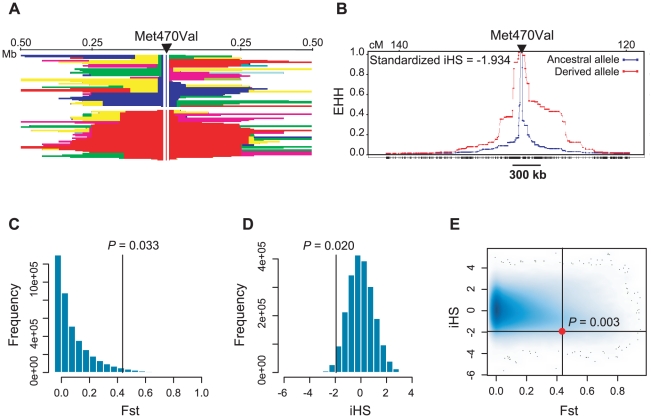
Population genetic parameters of the *CFTR* Met470Val locus. (A) Haplotype blocks +/− 500 kb around Met470Val locus in HapMap CEU (phase II) samples. The arrow indicates the location of Met470Val; the blue vertical line shows the ancestral Met470 allele and the red vertical line shows the derived Val470 allele. A continuous block of the same color represents the haplotypes shared between individuals. Haplotypes on the Met470 background are shorter and more variable compared to those on Val470 background. (B) Decay of extended haplotype homozygosity (EHH) around the Met470Val locus in the same data as in (A). The blue plot represents the decay of haplotypes on the ancestral (Met) allele background; the red plot represents the decay of haplotypes on the derived (Val) allele background. The Y-axis shows the EHH, defined as the probability that two randomly chosen chromosomes are homozygous at all SNPs for the entire interval from the core SNP at distance *x* [37]. EHH probability drops below 0.5 at approximately 300 kb around Met470Val on haplotypes carrying the Val470 allele, compared to <20 kb on haplotypes carrying the Met allele. The iHS corresponds to the natural logarithm of the ratio of areas under the ancestral and derived allele EHH curves, standardized to be independent of the allele frequencies. A negative iHS implies that the haplotypes on derived allele background are longer than those on ancestral background [23]. (C–E) Genome-wide distributions of (C) Fst values, (D) iHS scores and (E) Fst and iHS scores for SNPs in HapMap phase II data. Black lines (and filled circle in E) show the location of the Met470Val SNP in each distribution. Proportions of SNPs with more extreme values are shown on the plots as empirical *P*-values.

If the fertility effect associated with Met470Val genotypes in the Hutterites is generalizable, then the fitness advantage associated with the Val470 allele would be expected to leave a signature of positive selection on the pattern of variation at this locus [19]. Therefore, we examined Met470Val genotype data from the International HapMap Project [20] (http://www.hapmap.org/) and the Human Genome Diversity Project (HGDP) [21] (http://hagsc.org/hgdp/). The derived Val allele is very rare in sub-Saharan Africa (allele frequency ranges from 0 in Yorubans to 0.10 in Sans), whereas it occurs at high frequencies in non-African populations, and is even the more common allele in some European and Asian populations (reaching frequencies as high as 0.93 in Tuscans and 0.80 in Mongolians; Figure 3), as has been noted previously [22] and as we observed in the Amish. The differences in the allele frequency distributions are also reflected in HapMap samples, where the Fst between the European (CEU) and Yoruban (YRI) populations is 0.43 (compared to genome-wide average of 0.11). Moreover, extended haplotype homozygosity (EHH) in the CEU population is apparent on the Val background compared to the Met background (Figure 4A and 4B). The integrated haplotype score (iHS), a measure of EHH [23], is −1.93 (genome-wide average is 0). Compared to genome-wide distributions in HapMap Phase 2 data, an Fst of 0.43 falls in the upper 3.3% (CEU vs. YRI) and an iHS of −1.93 falls in the lower 2% (CEU) of SNPs (Figure 4C and 4D). The fraction of SNPs in these data with an Fst ≥0.43 and an iHS ≤−1.93 is 0.003 (Figure 4E). The combined observations of a high frequency derived Val allele outside of Africa, a high Fst value, and a long EHH on haplotypes carrying the Val allele are suggestive of positive selection, and is consistent with the advantageous fertility effects of the Val allele relative to the Met allele, as observed in this study.

Pompei et al. previously reported a lack of genetic variation in the *CFTR* gene in carriers of the Val470 allele in healthy Europeans sampled from six different geographical areas in Europe [24], and speculated that the Val470 allele was under positive selection by conferring an advantage in the presence of pathogenic diseases. While we can not rule out that the Val470 allele confers resistance to pathogens, our study provides support for an alternate hypothesis: the Val470 allele rose to high frequencies outside of Africa due to a fertility advantage in carrier men. The fact that this allele is either absent or very rare in African populations further suggests either that the allele arose after early humans left African or that there is additional (negative) selection on the Val470 allele in certain (African) environments.

In fact, given the large fertility effects observed in the Hutterites, it is surprising that the Val470 allele has not gone to fixation in non-African populations. However, there might be several reasons why this has not occurred. First, the combined data on fertility effects of the Val470 allele indicate that this allele can be associated with both increased and decreased fertility, depending on genetic background. In the presence of the 5T allele at the intron 8 polyT locus, Val470 increases the risk of CBAVD and male infertility [3]. In the absence of the 5T allele (as in the Hutterites), the Val470 allele is associated with increased male fertility relative to Met470. Although the mechanism of this interaction is obscure, it provides one example of counteracting variation that could increase the time to fixation of the Val470 allele. Second, as mentioned above, the Val allele could also be deleterious in certain environments, such as in the presence of specific pathogens or the 5T allele, as a result of its pleiotropic effects in other organ systems. Third, the fertility advantage we observed is restricted to males; we found no such association in Hutterite women (data not shown). This would further slow the spread of the allele as there would be no selection advantage in half of all Val carriers. Lastly, this study was conducted in a population living under optimal conditions for reproductive success, including excellent nutrition and abundant food, access to modern health care, and negligible maternal mortality. Thus, estimates of fitness effects based on Hutterite fertility rates are likely inflated compared to the effects in human populations throughout most of evolutionary history, when competing selective pressures were likely more prevalent. Taken together, the lack of fixation of the Val470 alleles in populations outside of African may not be inconsistent with the fertility effects observed in the Hutterites, but rather suggestive of antagonistic effects of other genetic variations or environment factors that tempered these effects during most of human evolution.

To our knowledge, this is the first report demonstrating that a common variation in the *CFTR* gene influences reproductive fitness in fertile, healthy men. Nearly all previous studies on *CFTR* mutations and reproduction in males have focused on patients with infertility. Increased prevalences of *CFTR* mutations in men with reduced sperm quality, with azoospermia without CBAVD, and with isolated CBAVD have been reported [1], suggesting the involvement of CFTR in sperm production and development [25]. Moreover, heterozygous *Cftr* ^+/−^ mice have reduced sperm fertilizing capacity and lower overall fertility [26]. Although little is understood about the physiological role of CFTR protein in the normal male reproductive system [27], it is known that the reproductive tissues are more sensitive to changes in CFTR function [3]. It is, therefore, possible that subtle differences in CFTR conductive properties between the Met and Val alleles may result in changes in the fluid environment of male reproductive tract, which would eventually lead to differences in sperm transport activity, morphology or quality [26], and could account for the observed fertility differences reported here. On the other hand, it is possible that the fertility effects of the Met470Val polymorphism described in this study are unique to the Hutterites and would not be replicated in other populations with measures of natural fertility and large family sizes. However, combined with the evolutionary signatures at this locus, the consistent (if not significant) results in the Amish, and the plausible biological mechanism, we believe that our data provide support for at least one specific variant in the *CFTR* gene influencing natural variation in fertility in healthy men.

Lastly, there has been a long-standing debate as to whether disease-causing CF mutations, such as ΔF508, confer a fertility advantage to healthy carriers (for example see Danks et al. [28]). Unfortunately, the results we report here do not provide insight into this question. The most common CF causing mutations in Europeans (i.e. ΔF508, G542X, N1303K, W1282X) and the most common mutation in the Hutterites, M1101K [16], all reside on haplotypes carrying the ancestral, Met470 allele in exon 10 [29], the 9T allele at the polyT locus, and (by inference) the TG10 or TG11 alleles at the (TG)m locus in intron 8 [5]. Therefore, any positive fertility effects of the Val470 allele would not be expected to affect the frequencies of the common CF disease-causing mutations in European populations.

In conclusion, the combined observations of high levels of variation in the *CFTR* gene, decreased fertility among CF patients and some CF carriers, and our observation of lower fertility associated with homozygosity for the Met470 allele in healthy men suggest that there are multiple independent, and possibly competing, evolutionary forces acting on the *CFTR* locus. The modifying effects of the haplotype background (i.e., 5T) on specific variants further imply important epistatic interactions between variants in the *CFTR* gene. Lastly, the high frequency of Val470 outside of Africa raises the possibility of interaction between *CFTR* alleles and changing environmental conditions. Thus, understanding the complex evolutionary history of the *CFTR* gene may require detailed studies of variation in worldwide samples of patients with CF and CF-related disorders, as well as healthy individuals. Regardless, this gene continues to provide surprises and represents outstanding examples of epistasis, in which the same allele (e.g., Val470) can have beneficial or deleterious effects depending on genetic background, and of a locus influenced by both positive (due to fertility advantage) and negative (due to CF and CF-related phenotypes) selection.

## Materials and Methods

### Ethics statement

Written consent was obtained from all participants before the studies. The study in the Hutterites was approved by the University of Chicago Institutional Review Board protocol (#5444). The study in the Amish was approved by the Institutional Review Board of the University of Maryland, Baltimore.

### Subjects and study design

The Hutterites are a young founder population that originated in the South Tyrol in the 16^th^ century, and migrated to the United States in the 1870s [9], [10]. The subjects of our study are related to each other through multiple lines of descent in a 13-generation pedigree consisting of 3,028 individuals, all of whom can be traced back to 62 founders [30]. We obtained birth, death and marriage dates from records compiled by the Hutterite ministers; reproductive history interviews were conducted in person by C.O. during field trips to Hutterite colonies between 1982 and 2007.

The Amish immigrated from central Europe (mainly Switzerland) to the United States to escape religious persecution over a 50-year period beginning in 1727 [31]. Members of the replication sample were enrolled in at least one of the studies at the University of Maryland, Baltimore beginning in 1996. Subjects were initially identified through prior participation in one of our studies, word of mouth, advertisements, a community-wide mailing, and referrals from local physicians. Reproductive health information, including the number and timing of births, were obtained from a self-reported questionnaire administered to female participants.

### Measure of fertility

We calculated interbirth intervals for each couple with two or more children. We defined “birth rate” as [(number of births – 1)/(sum of the interbirth intervals)]. Wife's birth year, which was highly correlated with husband's birth year (r^2^ = 0.98 in both the Hutterites and the Amish), and number of years from marriage to last birth were both significant predictors of birth rate, and were therefore included as covariates in a multivariate linear regression model. Residuals of birth rate obtained from this model were normally distributed, and used to test associations with *CFTR* polymorphisms. Details regarding the sample composition, heritability estimates, and distributions of fertility measures in the Hutterites are reported elsewhere [32].

### Genotyping and haplotype construction

Genotypes for Met470Val were obtained using a CFTR Linear Array platform from Roche Molecular Systems (Alameda, CA, USA) or TaqMan (ABI) in the Hutterites and Amish. Intron 8 polyT and (TG)m loci were genotyped by bidirectional sequencing of a single amplicon in the Hutterites only. Haplotypes at intron 8 could be unambiguously determined, as each diplotype produced a unique sequence pattern. In addition, because polyT and Met470Val polymorphisms were previously genotyped in the larger Hutterite pedigree, we constructed haplotypes by direct observations of alleles segregating in the families. Using these approaches, we were able to assign phase in 202 men; intron 8 sequence could not be obtained for one man, and phase could not be determined for one man who was heterozygous Met/Val and TG11/12, and homozygous 7T/7T.

### Statistical analyses

Associations in the Hutterites were tested using a regression-based test, designed for large complex pedigrees [17]. This method tests for associations under a general model, which allows for additive, dominant or recessive effects for each allele, and accounts for the relatedness between all pairs of individuals in our sample. *P*-values are corrected for multiple tests per SNP. In addition, we used a conservative Bonferroni correction to adjust for the multiple number of overall tests (n = 4; two models at the Val470 and one each at the (TG)m and polyT loci).

To estimate the effect size of an allele, we performed generalized linear regression, weighed by the estimated covariance matrix (obtained as described by Abney et al. [17]). Three models were tested at each locus. In the null model, the covariates were regressed against the phenotype; in the alternative models, genotype information at a locus was included as additional covariates, with additive and/or dominance effects. Significance was determined at each locus by an *F*-statistic. Percent variance explained is calculated by using the residual sum of squares (RSS) from each test, by the equation [(RSS_null_–RSS_alternative_)/RSS_null_]*100.

Statistical analysis in the Amish was performed using Mixed Model Analysis of Pedigrees (MMAP), in-house developed software (not published). In brief, we performed a measured genotype approach utilizing a t-test of the beta coefficient for the SNP variable. We included a polygenic component modeled as a random effect to account for the full 14 generation pedigree of the Amish.

We used nonparametric survival analysis to compare the distributions of the length of the interval from marriage to a specific birth among men with different genotypes [33], [34]. We selected the median and mean family size (N = 6 births) for this analysis and evaluated the time from marriage to 6^th^ birth. The interval lengths for men with fewer than 6 births at the time of our study were censored at the time of their last birth. The log-rank test was used to compare the time to 6^th^ birth curves between men with different genotypes. Statistical analyses were performed using JMP software (SAS Institute Inc., Cary, NC), version 7.0.1.

### Population genetic analyses

To examine the patterns of genetic variation around Met470Val locus, we used data from the International HapMap Project [20] (http://www.hapmap.org/) and the Human Genome Diversity Project (HGDP) [21] (http://hagsc.org/hgdp/). Fst values were estimated using Weir and Cockerham's theta [35], based on allele frequencies reported in HapMap Phase 2. Computation of a standardized iHS is explained in detail elsewhere [23]. Genome-wide distributions for Fst and iHS were generated for ∼3.1 million and ∼677,000 SNPs, respectively, in the HapMap Phase 2 data. Allele frequency distributions in HGDP were generated using HGDP Selection Browser website [http://hgdp.uchicago.edu/] [36]. Haplotype and EHH plots, and the standardized iHS presented in Figure 4A and 4B were obtained from Haplotter website (http://haplotter.uchicago.edu/selection/) [23].
